# Supplementary material for: Data-driven classification of tissue water populations by massively multidimensional diffusion-relaxation correlation MRI
Source: Front Neurosci. 2026 Mar 11;20:1716255. doi: 10.3389/fnins.2026.1716255 (PMC13013452; doi:10.3389/fnins.2026.1716255)
Supplement: Supplementary file 1 [file Data_Sheet_1.pdf]

Supplementary information:

Data-driven classification of tissue water populations by massively multidimensional diffusion-relaxation correlation MRI

Omar Narvaez[1], Maxime Yon[1,2], Raimo A. Salo[1], Jenni Kyyriäinen[1], Melina Estela[1], Ekaterina Paasonen[1,3], Ville Leinonen [4,5], Juhana Hakumäki [4,6], Frederik Laun[7], Daniel Topgaard[2] and Alejandra Sierra[1*]

[1] A.I. Virtanen Institute for Molecular Sciences, University of Eastern Finland, Kuopio, Finland, [2] Department of Chemistry, Lund University, Lund, Sweden, [3] Neurocenter, Kuopio University Hospital, Kuopio, Finland, [4] Institute of Clinical Medicine, University of Eastern Finland, Kuopio, Finland, [5] Neurocenter Neurosurgery, Kuopio University Hospital, Kuopio, Finland, [6] Department of Clinical Radiology, Kuopio University Hospital, Kuopio, Finland, [7] Institute of Radiology, University Hospital Erlangen, Erlangen, Germany

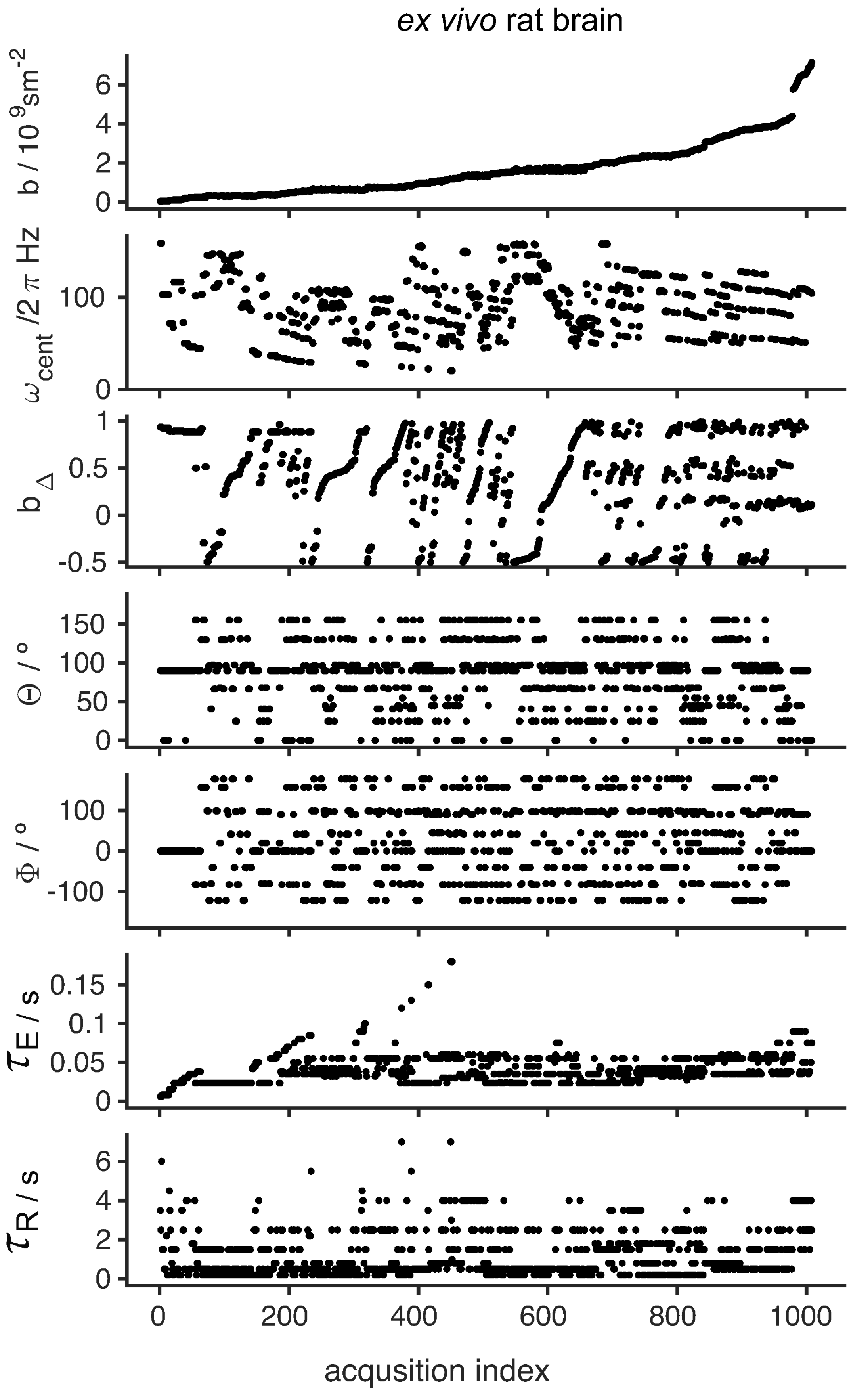

Supplementary Figure 1. Acquisition scheme for *ex vivo* rat brain.

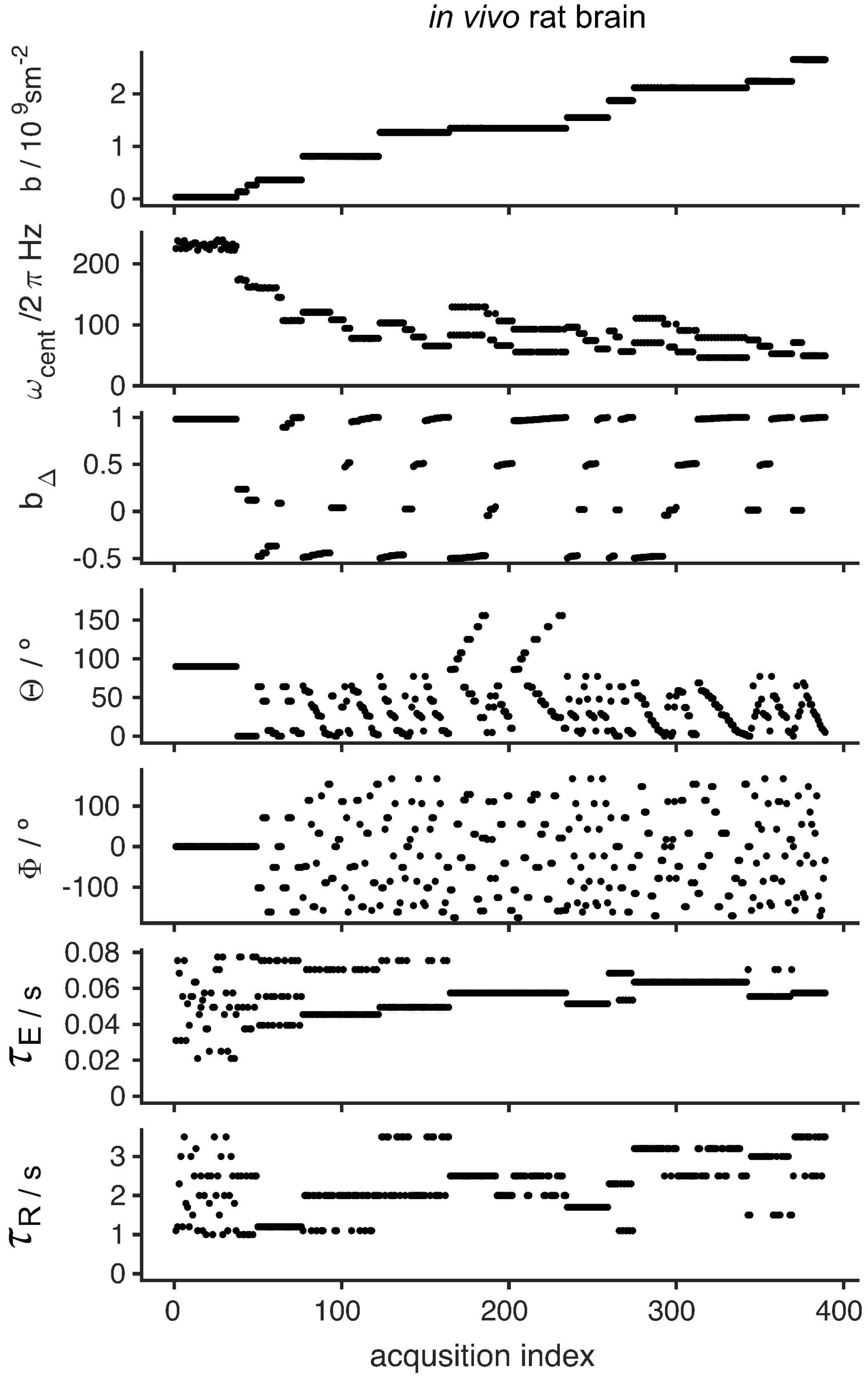

Supplementary Figure 2. Acquisition scheme for *in vivo* rat brain.

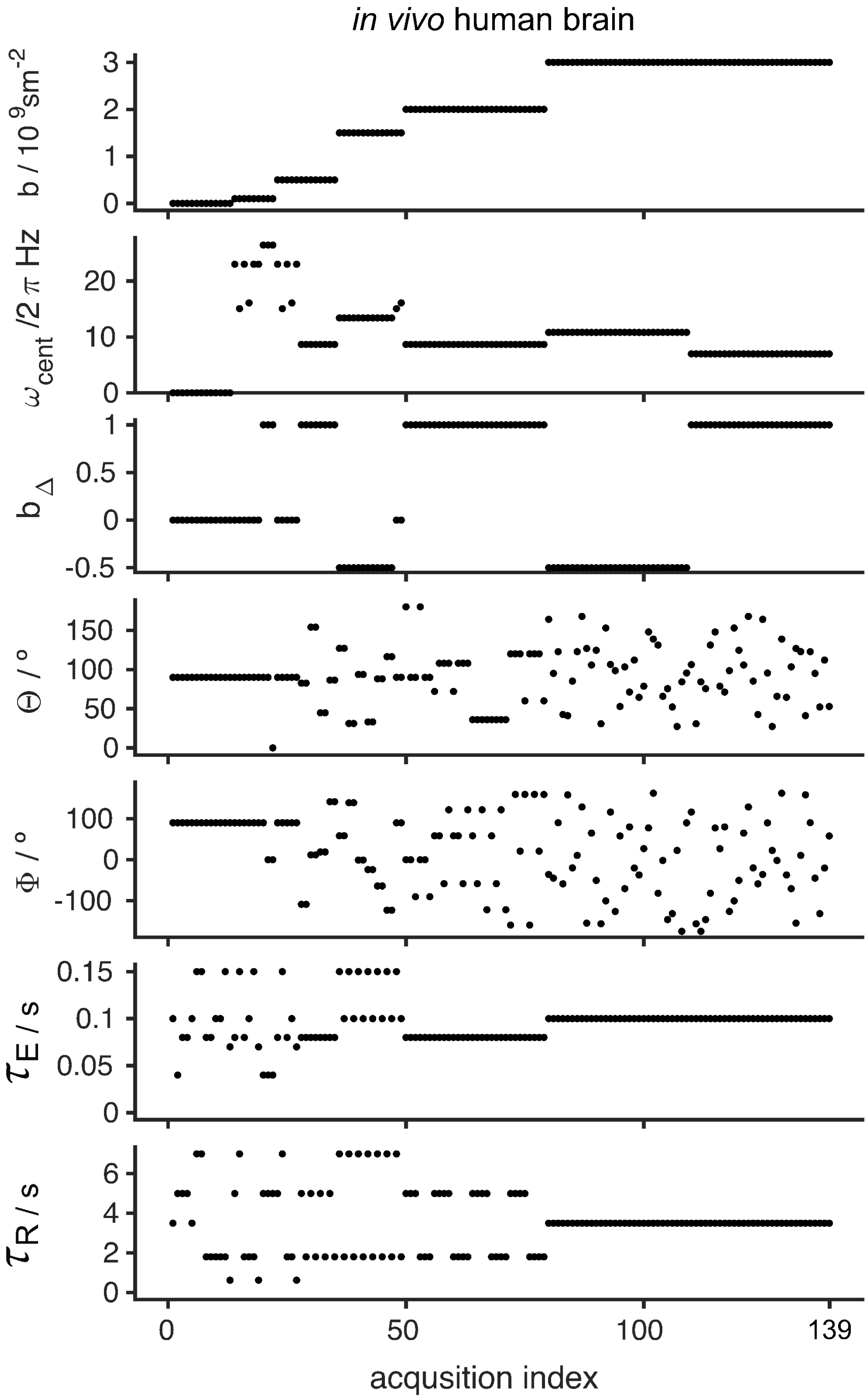

Supplementary Figure 3. Acquisition scheme for *in vivo* human brain.

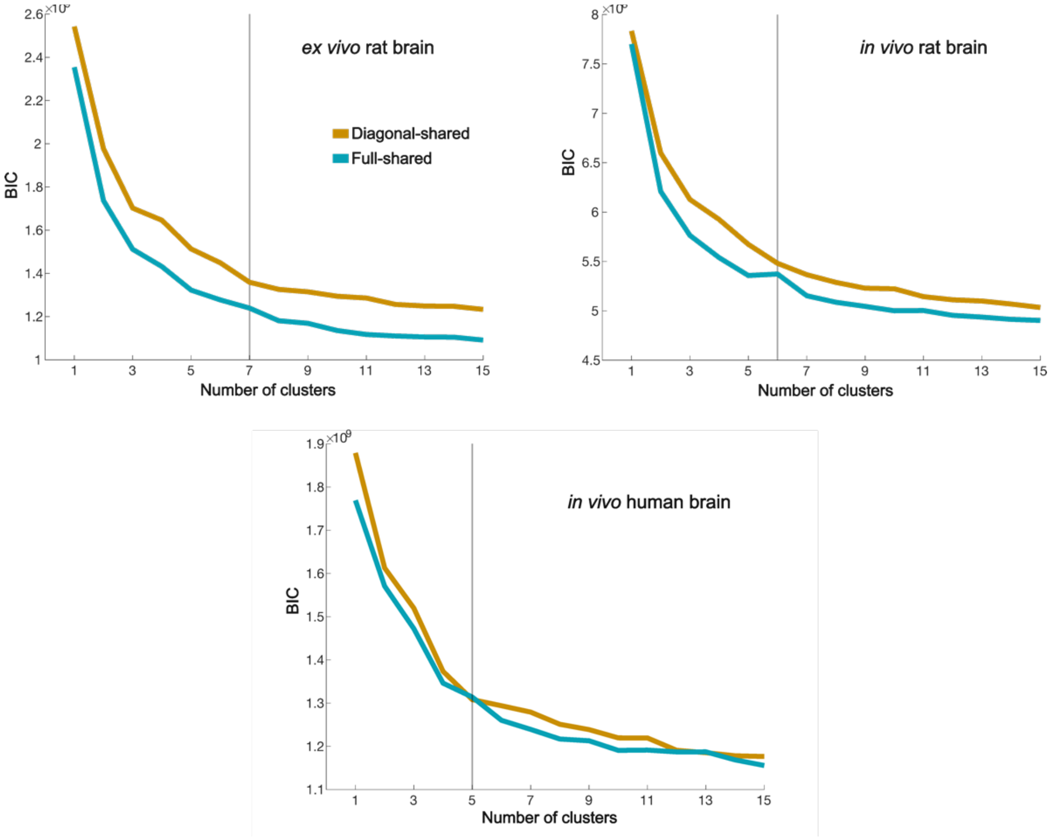

Supplementary Figure 4. Schwarz's Bayesian Information Criterion (BIC) from the clusterization of the D_{iso}, D_{Δ}, R_1, R_2 and $\Delta_{\omega/2\pi}D_{\mathrm{iso}}$ space of *ex vivo* rat brain, D_{iso}, D_{Δ}, R_1, R_2 and $\Delta_{\omega/2\pi}D_{\mathrm{iso}}$ space of *in vivo* rat brain, and D_{iso}, D_{Δ}, R_1, and R_2 space of *in vivo* human brain.

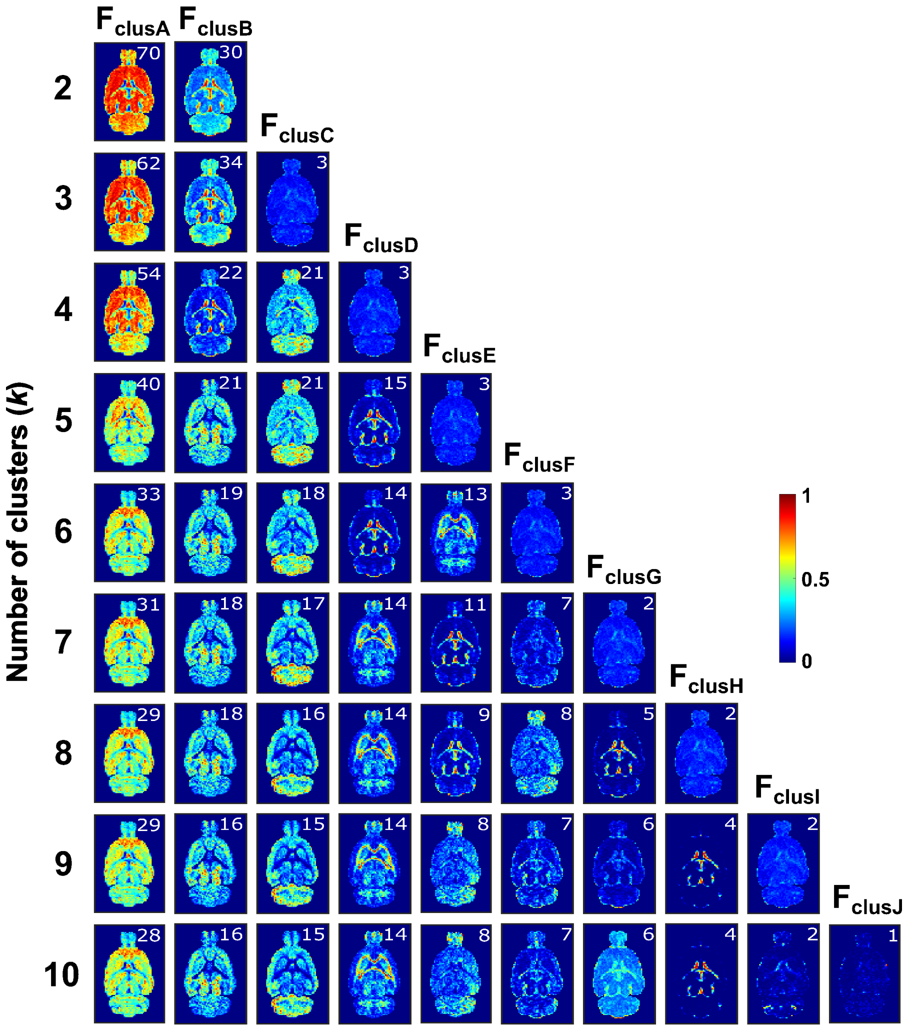

Supplementary Figure 5. Normalized cluster frequency of k = 2-10 from the full distribution space (D_{iso}, D_{Δ}, R_1, R_2 and $\Delta_{\omega/2\pi}D_{\mathrm{iso}}$) of *in vivo* rat brain data. Rows show the number of clusters and columns the normalized cluster frequency. The clusters are ordered by decreasing percentage of weight belonging to the cluster from left to right. The color scale shows the per-voxel weight belonging to each cluster.

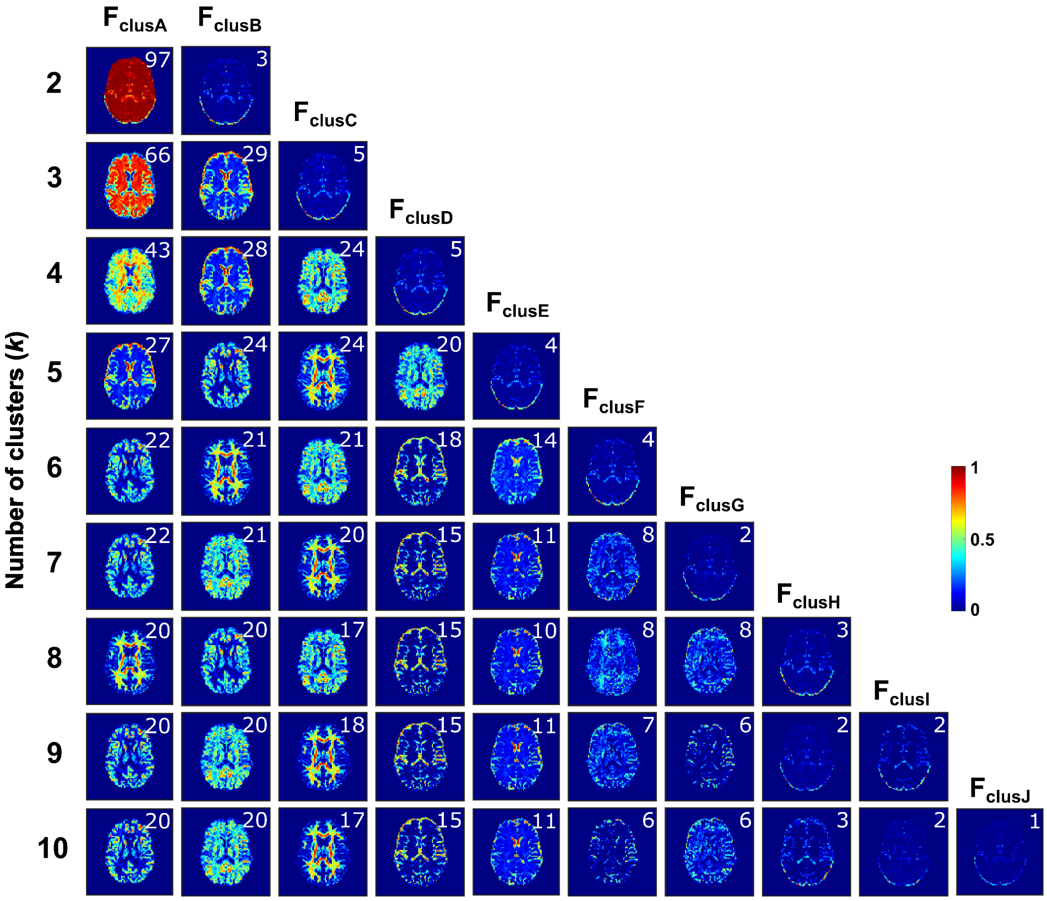

Supplementary Figure 6. Normalized cluster frequency of $k = 2\text{-}10$ from the D_{iso}, D_{Δ}, R_1, and R_2 distribution space of *in vivo* human data. Rows show the number of clusters and columns the normalized cluster frequency. The clusters are ordered by decreasing percentage of weight belonging to the cluster from left to right. The color scale shows the per-voxel weight belonging to each cluster.
